# Resting blood pressure reductions following handgrip exercise training and the impact of age and sex: a systematic review and narrative synthesis

**DOI:** 10.1186/s13643-018-0876-5

**Published:** 2018-12-12

**Authors:** Danielle C. Bentley, Cindy H. Nguyen, Scott G. Thomas

**Affiliations:** 1 Faculty of Medicine, University of Toronto, Toronto, Canada; 2 Faculty of Kinesiology and Physical Education, University of Toronto, Toronto, Canada

**Keywords:** Handgrip exercise, Systematic review, Blood pressure, Aging, Cardiovascular health, Cardiovascular risk

## Abstract

**Background:**

The risk of developing cardiovascular disease can be directly correlated to one’s resting blood pressure (BP), age, and biological sex. Resting BP may be successfully reduced using handgrip exercise training, although the impact of age and sex on training effectiveness has yet to be systematically evaluated. The objective of this systematic review is to determine this impact of age and sex on handgrip-induced changes to resting BP.

**Methods:**

Data sources included MEDLINE, Embase, Cochrane Reviews, CINAHL, SPORTDiscus, Web of Science, AMED, PubMed, and Scopus through May 2018. Eligibility criteria were those with prospective handgrip exercise training of ≥ 4 weeks with reported impact on resting systolic BP (SBP). Screening of articles, data extraction, and quality appraisal were completed in duplicate. When necessary, the corresponding authors were contacted to provide segregated data based on age (younger, 18–54 years; aged, > 55 years) and sex (men, women) categories. SBP was primarily explored with numerous secondary outcomes of interest summarized as a narrative synthesis.

**Results:**

After screening 1789 articles, 26 full texts were reviewed. Eight studies reported data in a way that facilitated age and sex comparisons of primary outcomes, while 7 of 18 studies reporting pooled data (men and women) provided segregated results. Research spans 1992–2018 and represents 466 participants; at least 43.1% of whom are women. Although weighted mean differences reveal that handgrip training-induced SBP reductions are similar when merely comparing sexes (women; − 5.6 mmHg, men; − 4.4 mmHg) or ages (younger; − 5.7 mmHg, aged; − 4.4 mmHg), when the impact of sex and age is simultaneously evaluated, aged women experience the largest reduction in SBP (− 6.5 mmHg). Many factors were explored for their impact on resting BP reductions and have been summarized in the corresponding narrative synthesis.

**Conclusions:**

Handgrip exercise is an effective modality for resting BP reduction resulting in clinically significant reductions for men and women of all ages.

**Systematic review registration:**

PROSPERO CRD42015019792

## Introduction

### Rationale

Cardiovascular diseases (CVD) are the leading cause of death worldwide, responsible for approximately 17.7 million (31%) of global deaths in 2015 [1]. There is a strong, independent correlation between CVD morbidity and mortality, and high blood pressure (BP) [2], with the maintenance of resting BP at an optimal level critical in order to reduce the global burden of CVD [3]. Emphasizing the importance of resting BP for CVD health, the recent Global Non-communicable Diseases action plan put forth by the World Health Organization calls for a 25% reduction in the global prevalence of raised BP (defined as systolic BP/diastolic BP > 140/> 90 mmHg) by 2025 [3]. Recent prevalence rates of elevated resting BP in adults have identified 24% of men and 20.1% of women [1, 3] over the age of 18 years, with this rate increasing to 63% of men and 67% of women over the age of 60 [4] .

The risk of developing CVD, most notably elevated resting BP, is influenced by both an individual’s age and their biological sex, with dissimilar patterns of incidence between the sexes. Women typically present with CVD 10 years later than men, with an exponential rise in CVD incidence rates aligning with the age of natural menopause [5]. A recent review summarized several primary studies that supported a direct relationship between the menopause transition and elevated resting BP [4], highlighting the impactful interaction of age and biological sex. Among Canadian women, the relative risk of developing CVD increases fourfold after the menopause transition [6]. Encouraging lifestyle modifications is a well-documented recommendation to combat the disproportionately low optimization of blood pressure among women [7].

To diminish the risk of CVD, it is recommended that individuals with above optimal BP engage in non-pharmaceutical interventions, such as exercise training [3, 8]. Although regular aerobic exercise (i.e., jogging, cycling) consistently reduces resting BP (− 3.5/− 2.5 mmHg) [9], barriers such as lack of exercise self-efficacy, physical limitations, and financial obstacles can limit uptake and adherence. An alternative to traditional aerobic exercise is handgrip exercise (both isometric and rhythmic prescriptions), which is easily accessible, requires little time, and may serve to introduce exercise behaviors to reluctant individuals. The American Heart Association’s (2013) scientific statement on alternative, non-pharmaceutical, approaches to lowering BP assigned isometric exercise training a class IIB level of evidence C recommendation, identifying a need for more research in the field with broader populations [10]. A recent meta-analysis on the topic of isometric resistive exercise literature identified six isometric handgrip studies revealing significant reductions in resting systolic BP (SBP) (mean; confidence interval: − 6.88; − 8.31 to − 5.46 mmHg) [11]. However, the small number of eligible studies using handgrip exercise precluded statistical assessments for the potential impacts of age and sex resting BP reduction outcomes. This limitation has also been encountered among previous literature reviews of isometric exercise which also concluded strong overall effectiveness without conducting sub-analyses of age or sex groups [2, 12–15]. Therefore, a systematic review of handgrip exercise training and its impact on resting BP reduction, with data segregated based on individual participant’s age and sex, is required to more comprehensively understand the training-induced impact on resting BP. The inclusion of diverse handgrip exercise designs (isometric and rhythmic) and the age- and sex-dependent assessments of secondary variables of cardiovascular health would further strengthen such a review.

### Objectives

Using a broad set of eligibility criteria and an inclusive search strategy this systematic review sought to assess the potential impacts of participants’ age and sex on the effectiveness of handgrip exercise training for resting SBP reduction. To further explore handgrip-induced SBP reductions secondary outcomes of interest include proposed correlates of handgrip-exercise-induced BP change.

## Methods

### Protocol and registration

Detailed eligibility criteria and methods of analysis were specified in advance and documented in a published review protocol [16]. This review conforms to the Preferred Reporting Items for Systematic Review and Meta-Analysis (PRISMA) guidelines [17] and is registered with the International Prospective Register of Systematic Reviews (PROSPERO) (registration number CRD42015019792). Ethical approval was not required.

### Eligibility criteria

Studies were eligible if they employed a handgrip training intervention of ≥4 weeks, without limitation on intervention design features (i.e., prescribed grip intensities, lengths of each individual grip contraction, training frequencies). A broad range of study designs were eligible, including randomized controlled trials (RCTs) with both inactive control groups (designated as “RCT – traditional”) and sham exercise control groups (designated as “RCT – sham”), and experimental exercise interventions without a designated control group (designated as “cohort”). Studies were excluded if they were retrospective, case series, or case reports. Research participants had to be adult humans of ≥ 18 years of age, although there were no limitations on participant comorbidities (i.e., hypertension, heart failure, diabetes) or medication use. Studies had to report the effect of handgrip exercise training on resting SBP. Studies that did not report the pre- to post-intervention change score in SBP or did not provide the necessary information for the reviewers to extract such information (i.e., those reporting mean arterial pressure (MAP) only) were deemed ineligible. Included studies may have also assessed the impact of handgrip training on a variety of additional cardiovascular assessments, as a primary, secondary, or tertiary outcome. Articles were included if they were published in English, Portuguese, or French.

### Information sources and search strategy

Based on the study eligibility criteria, a search strategy was developed consisting of a systematic, computer-assisted, literature search of existing evidence from several online databases: MEDLINE, Embase, Cochrane Reviews, Cumulative Index to Nursing and Allied Health Literature (CINAHL), SPORTDiscus, Web of Science, the Allied and Contemporary Medicine (AMED), PubMed, and Scopus. The search included articles published up to May 15, 2018. To ensure literature saturation, reference lists from relevant published reviews as well as retrieved articles were hand-searched and additional papers assessed for eligibility.

This review did not apply search limitations on study design, date, or language. When available, search limits were used on the variables of AGE (“adult < 18–64” and “aged < 65+>”) and TYPE (“human”). The search strategy was designed in conjunction with a University of Toronto research librarian with expertise in systematic reviews and was kept purposefully broad to increase the opportunity to identify potentially relevant papers. A representative OVID keyword search transcript is presented here which was applied to the databases of MEDLINE, Embase, and AMED.

1. [handgrip] OR [isometric grip] OR [static grip] OR [forearm grip]

2. [training] OR [intervention*] OR [exercise*] OR [physical activity]

3. [blood pressure] OR [systolic] OR [cardiovascular]

4. [1] AND [2] AND [3]

### Study selection

Following the removal of duplicates, two reviewers (DB and CN) independently screened the titles and abstracts of all papers identified through the search described above. Eligibility was tracked using a customized and standardized screening tool and EndNote (Version 5.0 Thompson Reuters, 2011). Papers that did not meet eligibility criteria were excluded, with discrepancies between authors resolved by consensus and consultation with a third reviewer (ST). Review authors were not blinded to the names or institution of study authors or to journal titles.

### Data collection process

Data extraction for all variables was independently completed by DB and CN using a customized data abstraction framework, with discrepancies assessed by ST. When required, corresponding authors of studies reporting results of mixed-age and/or mixed-sex cohorts were directly contacted to obtain segregated data for our desired sub-analyses on age (young vs. aged) and sex (women vs. men).

### Data items

Full-data extraction occurred for study design details (i.e., year of publication, country of research, exercise prescription, bilateral or unilateral exercise, training stimulus quantified as a total tension-time product (TTP); a calculation of effort using tension (in percent of maximal volitional contraction (MVC)) and time (in seconds) such that TTP = Time (second) x Tension (%MVC)), participant details (i.e., age (segregated for younger (< 55 years) and aged (≥ 55 years)), sex (segregated for male and female), resting BP status (normotensive (< 120/< 80 mmHg), above optimal (> 121/> 81 mmHg)), medication use, and comorbidities), primary cardiovascular variables (i.e., impact of handgrip training on BP, heart rate (HR), arterial health and function measurements, venous health, and function measurements), and any exercise training adherence information (i.e., number of dropouts, compliance to exercise protocol). When appropriate, weighted mean differences were calculated as weighted mean *= Σ* (*n*)(*Δ*)/*Σ* (*n*), where *Σ* = sum of all, *n* = sample size, and *Δ* = change scores. In addition, location of exercise (primarily at home, primarily in the laboratory), level of supervision (digital grip output/ in-laboratory supervision, grip device without feedback/unsupervised), and method of BP assessment (resting, ambulatory) were extracted.

The assumption was made that women in the “aged” category (≥ 55 years) were post-menopausal. Although natural menopause in women can be affected by a variety of factors such as ethnicity, diet, physical activity, and genetics [18], the National Institute of Aging states that the most recent average age of menopause is 51 [19].

Our primary outcome of interest was change in SBP associated with handgrip exercise training, measured as either resting or ambulatory.

Secondary outcomes of interest were collected and analyzed if sufficiently reported. Prospectively, they included additional indictors of cardiovascular health as well as proposed correlates of handgrip-induced BP change:

- Change in resting diastolic blood pressure (DBP)
- Change in HR
- Change in arterial health and function assessed via pulse wave velocity, arterial distensibility, reactive hyperamic forearm blood flow, or flow mediated vasodilation
- Change in venous health and function assessed via venous compliance
- Change in autonomic nervous system indicators, assessed via muscle sympathetic nerve activity (MSNA), heart rate variability (HRV), BP variability, or cardiovascular (CV) reactivity

### Risk of bias in individual studies

It was anticipated that included studies would have various research designs, some with and some without a randomized control group. Furthermore, exercise training studies have unique research design limitations regarding group allocation and blinding procedures. To ensure quality criteria specific to exercise training studies were appropriately evaluated the Tool for the assEssment of Study qualiTy and reporting in EXercise (TESTEX) was employed for quality assessment of the exercise interventions [20]. Using the TESTEX rubric, 12 criteria were evaluated with a maximum point allotment of 15; 5 points for study quality and 10 points for reporting.

### Summary measures and additional analyses

A narrative summary of all study design features, participant details, descriptive cardiovascular variables, and exercise training adherence information are presented. Changes to resting SBP have been calculated as weighted mean (weighted by sample sizes) differences, segregated by participants’ age and sex. Primary sub-analyses were conducted to determine the impact of age (younger vs. aged) and the impact of sex (women vs. men) on handgrip training-induced changes to resting SBP change. Additional sub-analyses explored the impact of participant characteristics (i.e., resting BP status at commencement of exercise training), exercise characteristics (i.e., handgrip force prescription, length of training, type of tool used), and location of exercise training (i.e., at home or in laboratory).

## Results

### Study selection

**Fig. 1:**
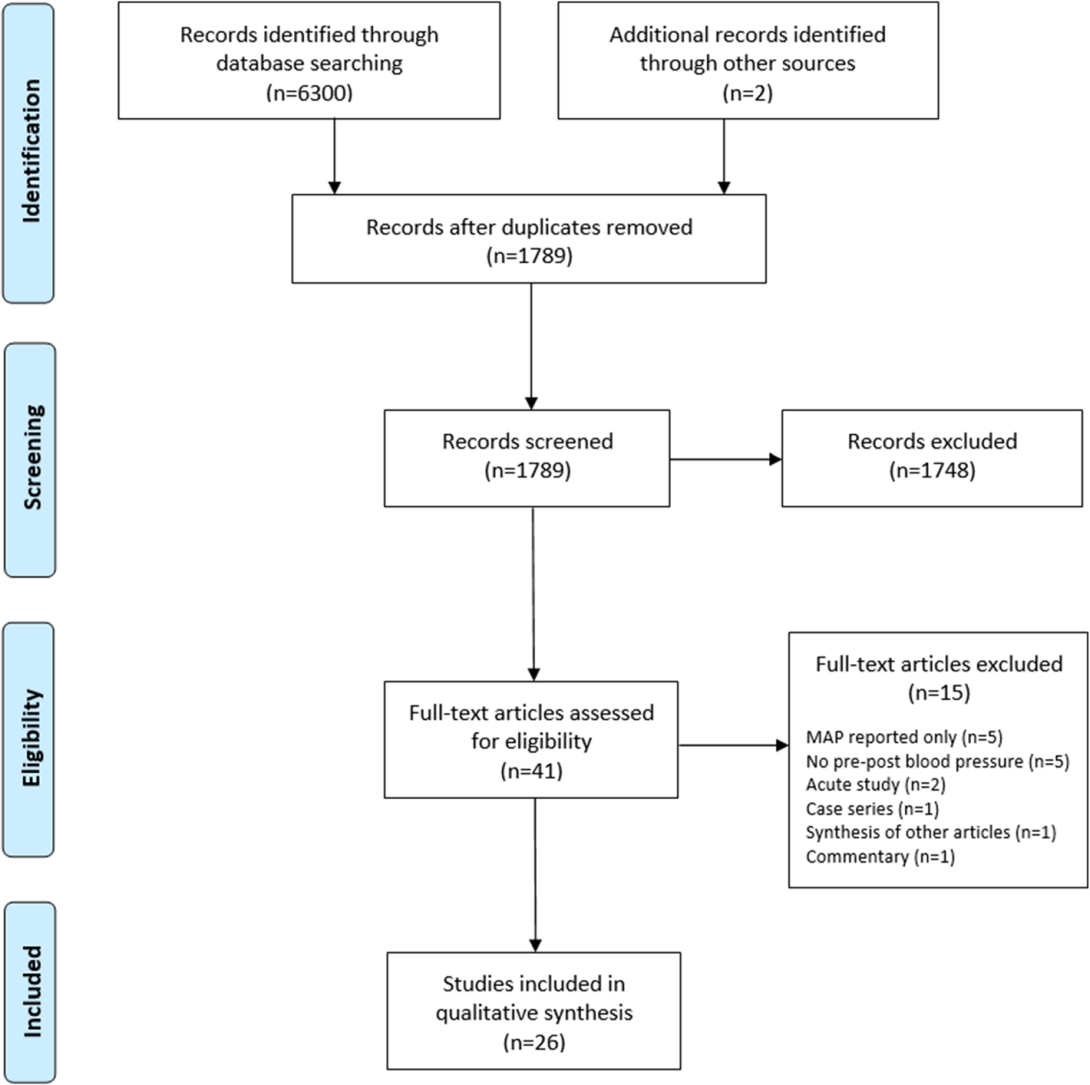
PRISMA flow diagram

Following the removal of duplicates, 1789 studies were screened (titles and abstracts) down to 41 potentially eligible studies. Full-text review led to the removal of 15 articles for reasons of not reporting pre- to post-intervention change in blood pressure [21–25], research was an acute assessment of handgrip-induced real-time fluctuations to BP [26, 27], research was a case study [28], research was a synthesis of other articles [29], publication was a commentary [30], and only MAP was reported with no way of extracting SBP and diastolic BP (DBP) [31–35]. Therefore, 26 studies were included in this review (Fig. 1).

### Study characteristics

Characteristics of included studies are comprehensively presented in Table 1.

**Table 1:** Study characteristics and main results of included studies

Author and country	Study design	Participants	Relevant comorbidities	Exercise frequency and duration	HG training prescription	Total training tension-time product	SBP training response	Secondary outcomes reported	Risk of bias (TESTEX)
Allen et al. [59] USA	Cohort	Men (*n* = 14), young (26 ± 5.7 years)		5/week for 4 weeks	One contraction every 4 s unilateral (non-dom) at 60% MVC for 20 min	360,000	− 4 mmHg (non-sig)	Diastolic BP, Heart rate, Arterial health and function (FMD), Autonomic Nervous system indicators (HRV)	7/15
Alomari et al. [60] no country noted	Cohort	Men (*n* = 11), young (24 ± 4.8 years)		5/week for 4 weeks	300 × 2 s unilateral (non-dom) HG contractions at 60% MVC, separated by 2-s rest	720,000	− 2 mmHg (non-sig)	Diastolic BP, Heart rate, Arterial health and function (reactive hyperamic blood flow + vascular resistance)	5/15
Badrov et al. [45] Canada	RCT—traditional	Men (*n* = 6), aged (67 years), Women (*n* = 6), aged (63 years)	All hypertensive and/or receiving pharmacotherapy for at least 4 months	3/week for 10 weeks	4 × 2 min bilateral HG contractions at 30% MVC, separated by 1-min rest	432,000	Men: − 7 mmHg (grouped change sig), Women: − 9 mmHg (grouped change sig)	Diastolic BP, Heart rate, Autonomic nervous system indicators (CV reactivity)	11/15
Badrov et al. [38] Canada	RCT—traditional	Women (*n* = 12), young (23 ± 4 years)		3/week for 8 weeks	4 × 2 min unilateral (non-dom) HG contractions at 30% MVC, separated by 4-min rest	345,600	− 6 mmHg (sig)	Diastolic BP, Heart rate, Autonomic nervous system indicators (HRV), Arterial health and function (reactive hyperamic blood flow)	13/15
		Women (*n* = 11), young (27 ± 6 years)		5/week for 8 weeks	4 × 2 min unilateral (non-dom) HG contractions at 30% MVC, separated by 4-min rest	576,000	− 6 mmHg (sig)		
Badrov et al. [54] Canada	Cohort	Men (*n* = 9), young (21 ± 2 years), Women (*n* = 11), young (23 ± 4 years)		3/week for 8 weeks	4 × 2 min unilateral (non-dom) HG contractions at 30% MVC, separated by 4-min rest	345,600	Men: − 9 mmHg (sig), Women: − 6 mmHg (sig)	Diastolic BP, Arterial health and function (FMD)	7/15
Baross et al. [44] * United Kingdom	RCT—traditional	Men (*n* = 6) and women (*n* = 16), young (20.7 ± 1.6 years)		4/week for 6 weeks	3 × 10 s bilateral HG contractions at 20% MVC, separated by 10-min rest	14,400	− 4.9 mmHg (sig)	Diastolic BP	6/15
Bentley et al. [49] Canada	Cohort	Women (*n* = 17), aged (61.6 ± 4 years)	At least pre-hypertensive or receiving pharmacotherapy	4/week for 8 weeks	32 × 5 s unilateral (non-dom) HG contractions at max MVC, separated by 5-s rest	512,000	− 5.2 mmHg (sig)	Diastolic BP, Heart rate, Autonomic nervous system indicators (HRV and CV reactivity), Arterial health and function (PWV and AIx)	9/15
Carlson et al. [41] * Australia	RCT—“sham”	Men and women (*n* = 18), aged (52 ± 8 years)	At least pre-hypertensive or receiving pharmacotherapy	3/week for 8 weeks	4 × 2 min unilateral (non-dom) HG contractions at 30%MVC, separated by 3-min rest	345,600	− 7 mmHg (sig)	Diastolic BP, Heart rate	11/15
		Men and women (*n* = 20), aged (54 ± 8 years)	At least pre-hypertensive or receiving pharmacotherapy	3/week for 8 weeks	4 × 2 min unilateral (non-dom) HG contractions at 5%MVC, separated by 3-min rest	57,600	− 2 mmHg (non-sig)		
Dawson et al. [42] no country noted	RCT—traditional	Men (*n* = 8), aged (65 years), Women (*n* = 1), young (47 years)	All patients required transradial catheterization for upcoming coronary angiography/angioplasty	3/week for 6 weeks	900 × 1 s unilateral (catheterized arm) HG contractions at 40% MVC, separated by 1-s rest	648,000	Men: + 1 mmHg (grouped change non-sig), Women: + 16 mmHg (grouped change non-sig)	Heart rate, Arterial health and function (FMD)	5/15
Dobrosielski et al. [51] USA	Cohort	Men (*n* = 12), aged (81 ± 5 years)		4/week for 4 weeks	300 × 1 s unilateral (non-dom) HG contractions at 60% MVC, separated by 3-s rest	288,000	− 2 mmHg (non-sig)	Diastolic BP, Heart rate, Arterial health and function (brachial artery reactivity and FMD)	8/15
Garg et al. [39] * India	Cohort	Men (*n* = 16) and women (*n* = 14), young (29.8 ± 6.3 years)		3/week for 10 weeks	5 × 3 min unilateral (dominant) HG contractions at 30% MVC, separated by 5-min rest	810,000	− 9.87 mmHg (sig)	Diastolic BP	3/15
Goessler et al. [52] * Belgium	RCT—traditional	Men and women (*n* = 19), young (33.1 ± 2.1 years)		7/week for 8 weeks	4 × 2 min bilateral contractions at 30% MVC, separated by 1-min rest	806, 400	− 5.5 mmHg (sig)	Diastolic BP	8/15
Hess et al. [61] * Australia	RCT—“sham”	Men (*n* = 6) and women (*n* = 4), young (38.8 ± 10.5 years)		3/week for 6 weeks	4 × 2 min unilateral (non-dom) contractions at 5% MVC, separated by 1-min rest	43,200	− 4.04 mmHg (non-sig)	Diastolic BP, Heart rate	11/15
		Men (n = 6) and Women (n = 4), young (38.7 ± 12.6 years)		3/week for 6 weeks	4 × 2 min unilateral (non-dom) contractions at 10% MVC, separated by 1-min rest	86,400	− 5.62 mmHg (non-sig)		
Kumar et al. [39] United Kingdom	Cohort	Men (*n* = 10), aged (avg: 63 years), Women (*n* = 13), aged (avg: 66 years)	Stable (stage 3–4) chronic kidney disease	7/week for 4 weeks	600 × 1 s unilateral (self-selected arm) HG contractions at max effort, separated by 2-s rest	1,680,000	Men: − 2.2 mmHg (grouped change non-sig) Women: 0 mmHg (grouped change non-sig)	Diastolic BP, Arterial health and function (brachial and radial resting diameters)	7/15
McGowan et al. [55] Canada	Cohort	Men (*n* = 8), young (avg: 23 years), Women (*n* = 3), young (avg: 39 years)		3/week for 8 weeks	4 × 2 min unilateral (non-dom) HG contractions at 30% MVC, separated by 4-min rest	345,600	Men: − 6 mmHg (grouped change sig) Women: − 3 mmHg (grouped change sig)	Diastolic BP, Arterial health and function (FMD)	9/15
McGowan et al. [46] Canada	Cohort	Men (*n* = 12), aged (avg: 63 years)	Medicated hypertensive	3/week for 8 weeks	4 × 2 min unilateral (non-dom, *n* = 9) or bilateral (*n* = 7) HG contractions at 30% MVC, separated by 4-min rest	345,600	− 12 mmHg (grouped change sig)	Diastolic BP, Arterial health and function (FMD)	9/15
		Women (*n* = 4), aged (avg: 68)					− 13 mmHg (grouped change sig)		
Millar et al. [47] Canada	Cohort	Men (*n* = 11), aged (avg: 66 years), Women (*n* = 2), aged (avg: 73 years)	Medicated Hypertensive	3/week for 8 weeks	4 × 2 min unilateral (non-dom) HG contractions at 30% MVC, separated by 4-min rest	345,600	Men: − 2 mmHg (grouped change sig) Women: − 15 mmHg (grouped change sig)	Diastolic BP, Heart rate, Autonomic nervous system indicators (HRV)	9/15
Millar et al. [53] Canada	RCT—traditional	Men (*n* = 14), aged (avg: 68 years), Women (*n* = 11), aged (avg: 63 years)		3/week for 8 weeks	4 × 2 min bilateral HG contractions at 30–40% MVC, separated by 1-min rest	345,600–460,800	Men: − 7 mmHg (grouped change sig) Women: − 11 mmHg (grouped change sig)	Diastolic BP	4/15
Pagonas et al. [50] * Germany	RCT—“sham”	Men and women (*n* = 24), aged (58.8 ± 10.6 years)	Hypertensive	5/week for 12 weeks	4 × 2 min bilateral HG contractions at 30% MVC, separated by 1-min rest	864,000	0 mmHg	Diastolic BP, Arterial health and function (PWV)	8/15
		Men and women (*n* = 23), aged (62.1 ± 7.1 years)	Hypertensive	5/week for 12 weeks	4 × 2 min bilateral HG contractions at 5% MVC, separated by 1-min rest	144,000	+ 1.4 mmHg (non-sig)		
Peters et al. [37] * USA	Cohort	Men (*n* = 8) and women (*n* = 2), aged (52 ± 5)	Hypertensive	3/week for 6 weeks	4 × 45 s bilateral HG contractions at 50% MVC, separated by 1-min rest	162,000	− 13 mmHg (sig)	Diastolic BP	3/15
Ray et al. [56] * USA	RCT—traditional	Men and women (*n* = 9), young (19 to 35 years)		4/week for 5 weeks	4 × 3 min unilateral (non-dom) contractions at 30% MVC separated by 5-min rest	432,000	− 3 mmHg (non-sig)	Diastolic BP, Heart Rate, Autonomic Nervous System Indicators (MSNA)	6/15
Somani et al. [40] Canada	Cohort	Men (*n* = 13), young (24 ± 4 years), Women (*n* = 13), young 25 ± 5 years		3/week for 10 weeks	4 × 2 min bilateral HG contractions at 30% MVC, separated by 1-min rest	432,000	Men: − 5 mmHg (sig) Women: − 3 mmHg (sig)	Diastolic BP, Heart rate, Autonomic nervous system indicators (CV reactivity)	10/15
Stiller-Moldovan et al. [48] * Canada	RCT—traditional	Men (*n* = 7) and women (*n* = 3), aged (60 ± 8.5 years)	Medicated hypertensive	3/week for 8 weeks	4 × 2 min bilateral HG contractions at 30–40% MVC, separated by 1-min rest	345,600	− 1.9 mmHg (non-sig)	Diastolic BP, Heart rate, Autonomic nervous system indicators (HRV)	10/15
Taylor et al. [43] * Canada	RCT—traditional	Men (*n* = 5) and women (*n* = 4), aged (69.3 ± 6 years)	Hypertensive	3/week for 10 weeks	4 × 2 min bilateral HG contractions at 30% MVC, separated by 1-min rest	432,000	− 19 mmHg (sig)	Diastolic BP, Heart rate, Autonomic nervous system indicators (HRV)	6/15
Thijssen et al. [58] United Kingdom	Cohort	Men (*n* = 11), young (22 ± 2 years)		4/week for 8 weeks	30 contractions/min bilateral contractions for 30 min at 30% MVC for 4 weeks, 40% MVC for 2 weeks and 50% MVC final 2 weeks	1,080,000	0 mmHg (non-sig)	Diastolic BP, Heart rate, Arterial health and function (wall-to-lumen ration)	7/15
Wiley et al. [57] * USA	Cohort	Men or women (*n* = 8), young (20–35 years)		3/week for 8 weeks	4 × 2 min unilateral (dom) contractions at 30% MVC separated by 3-min rest	345,600	− 13 mmHg (sig)	Diastolic BP, Heart rate	6/15
		Men or women (*n* = 10), young (29 to 52 years)		5/week for 5 weeks	4 × 45 s bilateral contractions at 50% MVC, separated by 1-min rest	216,000	− 9.5 mmHg (sig)		

*MVC* maximal volitional contraction

*Unable to acquire raw data to segregate for age and/or sex

### Risk of bias within studies

TESTEX scores ranged from 3 [36, 37] to 13 [38] (out of a total 15 marks) with median score of 7.5/15. When sub-divided based on study design, RCT studies (both traditional and sham) scored a median of 8.0 while cohort studies scored a median of 7.0. The lowest scored categories were Blinding of Assessor [37, 39] and Intention-to-Treat Analysis [40, 41] each of which only two studies scored positively for. Total TESTEX scores can be found in Table 1.

### Synthesis of results

Heterogeneity across included literature was very high with training-induced changes in resting SBP ranging from + 16 mmHg [42] to − 19 mmHg [43]. The large range of training-induced reductions may reflect the diversity of participants’ sex, age, and general health status or may reflect the diversity of training stimulus as TTP scores ranged from 14,400(seconds)(%MVC) [44] to 1,680,000(seconds)(%MVC) [39]. As per our original design [16], this review has therefore progressed with a narrative synthesis.

#### Participant details

In total, this review represents 466 participants, at least 43% (*n* = 201) of which can be identified as women. Within the reviewed literature at least 35% (*n* = 9) of studies recruited pre-hypertensive individuals with or without concomitant health conditions [37, 41–43, 45–51]. Although challenging to individually identify, as many as 101 participants were taking various prescription medications for BP control [41–43, 45–51]. A sample of recruited clinical populations includes those with kidney disease [39], diabetes mellitus [49, 50], atrial fibrillation [50], and coronary artery disease [47, 50] and those about to undergo coronary angiography [42].

#### Study design details including exercise intervention information

Research spans from 1992 to 2018, with 65% of the reviewed work (*n* = 17) published from 2010 onwards. Eleven studies employed an RCT design, with either a non-exercise control (*n* = 8) or a sham exercise control (*n* = 3), while fifteen (58%) studies employed a cohort design. Nearly half of the research (*n* = 11) was conducted in Canada.

The majority of researchers prescribed moderate intensity handgrip training with four to five repetitions of 2 to 3-min handgrip contractions at 30% MVC either bilaterally with 1 min of rest between alternating hands [40, 43, 45, 46, 48, 50, 52, 53] or unilaterally with 1–5 min of rest between contractions [36, 38, 41, 46, 47, 54–57]. Other handgrip training designs were more rhythmic (intermittent) with grip durations of 1 to 10 s at either 20% MVC [44], 30% MVC [58], 40% MVC [42, 58], 50%MVC [37, 58], 60%MVC [51, 59, 60], or maximum effort [39, 49]. Three studies used “sham” exercise conditions consisting of 4 × 2 min at 5% MVC [41, 50, 61], which for the purpose of this review have been included as a training condition.

Training was most commonly prescribed for 8 weeks, with a range from 4 to 12 weeks. None of the studies reported any adverse events from handgrip exercise, including those researchers who enrolled clinical populations.

Total exercise training effort was calculated as TTP/session multiplied by the total number of sessions participants completed. Two studies had training methods that did not allow for exact TTP calculation [24, 53]. TTP scores ranged from 14,400(seconds)(%MVC) [44] to 1,680,000(seconds)(%MVC) [39] with an average of 689,600(seconds)(%MVC). The most common TTP was a score of 345,600(seconds)(%MVC) calculated from 4 × 2 min @ 30%MVC, 3/week for 8 weeks [38, 41, 46–48, 54, 55, 57].

Participant dropout was not reported in 62% of studies (*n* = 16) and, when reported, occurred specifically due to non-compliance (*n* = 6) [50, 57, 59], family circumstances (*n* = 3) [55, 61], voluntary withdrawal (*n* = 3) [52, 59], personal illness (*n* = 2) [38, 55], work commitments (*n* = 2) [41], extenuating circumstances (*n* = 2) [46], time restraints (*n* = 2) [48], unrelated hand injury [49], and pregnancy [52]. Participant dropout within at least one research group was reported as zero in eight studies [38, 41, 43, 44, 53, 54, 57, 58].

There were 12 studies that collected data on participant adherence to handgrip training prescriptions, all of which reported very high values of ≥ 90% [52, 58], ≥ 95% [40, 49, 50], and 100% [38, 41, 45, 47, 51, 54, 61].

While the majority of research designs required participants to complete exercise primarily in the laboratory under direct supervision of investigators, there were eight studies which prescribed primarily at-home/unsupervised training regimens [38, 42, 45, 49, 50, 52, 57], representing half of the aforementioned reports of high adherence [38, 40, 45, 49, 50, 52]. Location of exercise training was unclear from the other research designs.

#### Age and sex introduction

Just under half of the training groups studied younger participants (< 55 years of age; 19/40).

In order to adequately segregate the data by sex, authors reporting results of mixed men and women groups (*n* = 18) were contacted and segregated data was requested. Appreciatively, seven (~ 39%) provided such information and the data has been presented accordingly in Tables 1, 2, 3, and 4. The remaining studies with mixed data of both men and women were combined to create a third category for the inclusion of weighted mean calculations looking at the impact of age. However, those studies were excluded from the weighted mean calculation on the impact of sex. For two studies, the sex of the participants was not reported and so it was assumed that the cohort was of mixed men and women [56, 57].

**Table 2:** Calculated weighted mean differences for reported systolic blood pressure (mmHg)

	Young (< 55 years)	Aged (> 55 years)	
Men *n* = 139	− 2 mmHg (*n* = 11) [60] *− 6 mmHg* (*n* = 8) [55] 0 mmHg (*n* = 11) [58] − 4 mmHg (*n* = 14) [59] *− 9 mmHg* (*n* = 9) [54] *− 5 mmHg* (*n* = 13) [40]	*−7 mmHg* (*n* = 6) [45] − 2 mmHg (*n* = 12) [51] − 2.2 mmHg (*n* = 10) [39] *− 12 mmHg* (*n* = 12) [46] − *2 mmHg* (*n* = 11) [47] *− 7 mmHg* (*n* = 14) [53] + 1 mmHg (*n* = 8) [42]	Weighted mean (young and aged men): − 4.43 mmHg
	Weighted mean: − 4.12 mmHg (*n* = 66)	Weighted mean: − 4.71 mmHg (*n* = 73)	
Women *n* = 104	*−6 mmHg* (*n* = 12) [38] *− 6 mmHg* (*n* = 11) [38] *− 3 mmHg* (*n* = 3) [55] *− 6 mmHg* (*n* = 11) [54] + 16 mmHg (*n* = 1) [42] *− 3 mmHg* (*n* = 13) [40]	0 mmHg (*n* = 13) [39] *− 9 mmHg* (*n* = 6) [45] *− 13 mmHg* (*n* = 4) [46] *− 15 mmHg* (*n* = 2) [47] *− 11 mmHg* (*n* = 11) [53] *− 5.2 mmHg* (*n* = 17) [49]	Weighted mean (young and aged women): − 5.59 mmHg
	Weighted mean: − 4.63 mmHg (*n* = 51)	Weighted mean: − 6.52 mmHg (*n* = 53)	
Men + women *n* = 222 (at least 96 women)	*− 13 mmHg* (*n* = 8) [57] *− 9.5 mmHg* (*n* = 10) [57] − 4.04 mmHg (*n* = 10, 4 women) [61] *sham − 5.62 mmHg (*n* = 10, 4 women) [61] *− 9.87 mmHg* (*n* = 30, 14 women) [36] − 3 mmHg (*n* = 9) [56] − 4.9 mmHg (*n* = 12, 6 women) [44] *− 5.5 mmHg* (*n* = 18, 8 women) [52]	− 1.9 mmHg (*n* = 11, 4 women) [48] *− 19 mmHg* (*n* = 9, 4 women) [43] *− 7 mmHg* (*n* = 18, 11 women) [41] − 2 mmHg (*n* = 20, 12 women) [41] *sham *− 13 mmHg* (*n* = 10, 2 women) [37] 0 mmHg (*n* = 24, 15 women) [50] + 1.4 mmHg (*n* = 23, 12 women) [50] *sham	
	Weighted mean: −7.26 mmHg (*n* = 107)	Weighted mean: − 3.96 mmHg (*n* = 115)	
	Weighted mean (young men and women): −5.73 mmHg	Weighted mean (aged men and women): − 4.75 mmHg	

Italicized values represent statistically significant reductions, as per either the original published results or the data provided directly by authors. Decimal places as per the original published results or the data provided directly by authors

**Table 3:** Calculated weighted mean differences for reported diastolic blood pressure (mmHg)

	Young (< 55 years)	Aged (> 55 years)	
Men *n* = 139	− 0.2 mmHg (*n* = 11) [60] **−** 1 mmHg (*n* = 8) [55] + 1 mmHg (*n* = 11) [58] 0 mmHg (*n* = 14) [59] *− 2 mmHg* (*n* = 9) [54] 0 mmHg (*n* = 13) [40]	*−4 mmHg* (*n* = 6) [45] − 1 mmHg (*n* = 12) [51] − 0.9 mmHg (*n* = 10) [39] − 4 mmHg (*n* = 12) [46] 0 mmHg (*n* = 11) [47] *− 3 mmHg* (*n* = 14) [53] − 2 mmHg (*n* = 8) [42]	Weighted mean (young and aged men): − 1.21 mmHg
	Weighted mean: − 0.26 mmHg (*n* = 66)	Weighted mean: − 2.07 mmHg (*n* = 73)	
Women *n* = 104	− 3 mmHg (*n* = 12) [38] 0 mmHg (*n* = 11) [38] 0 mmHg (*n* = 3) [55] *− 3 mmHg* (*n* = 11) [54] + 8 mmHg (*n* = 1) [42] − 1 mmHg (*n* = 13) [40]	− 0.8 mmHg (*n* = 13) [39] *− 4 mmHg* (*n* = 6) [45] − 5 mmHg (*n* = 4) [46] − 11 mmHg (*n* = 2) [47] *− 2 mmHg* (*n* = 11) [53] − 1.7 mmHg (*n* = 17) [49]	
	Weighted mean: − 1.45 mmHg (*n* = 51)	Weighted mean: − 2.40 mmHg (*n* = 53)	Weighted mean (young and aged women): − 1.94 mmHg
Men + women *n* = 222 (at least 96 women)	*− 14.9 mmHg* (*n* = 8) [57] *− 8.8 mmHg* (*n* = 10) [57] − 0.97 mmHg (*n* = 10, 4 women) [61] + 1.8 mmHg (*n* = 10, 4 women) [61] *− 5.26 mmHg* (*n* = 30, 14 women) [36] *− 5 mmHg* (*n* = 9) [56] − 1.8 mmHg (*n* = 18, 8 women) [52] − 2.4 mmHg (*n* = 12, 6 women) [44]	− 1.6 mmHg (*n* = 1, 4 women) [48] − 7 mmHg (*n* = 9, 4 women) [43] − 2 mmHg (*n* = 18, 11 women) [41] − 3 mmHg (*n* = 20, 12 women) [41] − 0.7 mmHg (*n* = 24, 15 women) [50] 2.8 mmHg (*n* = 23, 12 women) [50] − 2 mmHg (*n* = 10, 2 women) [37]	
	Weighted mean: − 4.33 mmHg (*n* = 107)	Weighted mean: − 1.30 mmHg (*n* = 115)	
	Weighted mean (young men and women): − 2.47 mmHg	Weighted mean (aged men and women): − 1.77 mmHg	

Italicized values represent statistically significant reductions, as per either the original published results or the data provided directly by authors. Decimal places as per the original published results or the data provided directly by authors

**Table 4:** Calculated weighted mean differences for reported heart rate (bpm)

	Young (< 55 years)	Aged (> 55 years)	
Men *n* = 83	+ 0.1 bpm (*n* = 11) [60] 0 bpm (*n* = 11) [58] − 4 bpm (*n* = 14) [59] + 1 bpm (*n* = 13) [40]	− 2 bpm (*n* = 12) [51] + 2 bpm (*n* = 8) [42] + 1 bpm (*n* = 14) [53]	Weighted mean (young and aged men): − 0.59 bpm
	Weighted mean: − 0.86 bpm (*n* = 49)	Weighted mean: − 0.18 bpm (*n* = 34)	
Women *n* = 65	−1 bpm (*n* = 12) [38] 0 bpm (*n* = 11) [38] + 14 bpm (*n* = 1) [42] − 3 bpm (*n* = 13) [40]	− 1.1 bpm (*n* = 17) [49] 0 bpm (*n* = 11) [53]	Weighted mean (young and aged women):− 0.86 bpm
	Weighted mean: − 1.0 bpm (*n* = 37)	Weighted mean: − 0.67 bpm (*n* = 28)	
Men + Women *n* = 130 (at least 47 women)	− 2 bpm (*n* = 8) [57] 0 bpm (*n* = 10) [57] 0 bpm (*n* = 10, 4 women) [61] 0 bpm (*n* = 10, 4 women) [61] − 1 bpm (*n* = 9) [56]	− 0.7 bpm (*n* = 11, 4 women) [48] − 2 bpm (*n* = 9, 4 women) [43] − 2 bpm (*n* = 13, 2 women) [47] 0 bpm (*n* = 12, 6 women) [45] + 2 bpm (*n* = 18, 11 women) [41] − 1 bpm (*n* = 20, 12 women) [41]	
	Weighted mean: − 0.53 bpm (*n* = 47)	Weighted mean: − 0.43 bpm (*n* = 83)	
	Weighted mean (young men and women): − 0.59 bpm	Weighted mean (aged men and women): − 0.33 bpm	

Italicized values represent statistically significant reductions, as per either the original published results or the data provided directly by authors. Decimal places as per the original published results or the data provided directly by authors

If the authors published the phrase “non-significant changes” in their reported [57, 36] then a value of “0” was entered and analyzed

#### Main outcome (SBP) and the impact of age and sex

Nearly all of the BP data presented and analyzed represents discrete readings taken in-lab, with only one research group only ambulatory BP [48]. Summaries of SBP change can be found in Table 2.

Weighted mean difference calculations reveal that handgrip training-induced SBP reductions were similar when comparing sexes (women, − 5.6 mmHg; men, − 4.4 mmHg) or ages (younger, − 5.7 mmHg; aged, − 4.4 mmHg). However, when the interaction effect of sex and age is simultaneously evaluated, aged women experience the largest handgrip training-induced reduction in SBP (− 6.5 mmHg).

##### Planned sub-analyses and the impact on SBP reduction

The resting BP status of participants at commencement of exercise training was explored as a correlate of resting SBP change, using the average resting BP reported within the original research. Interestingly, all research which exclusively recruited hypertensive individuals utilized moderate intensity handgrip exercise consisting of 4 × 2 min contractions at 30–40%MVC 3/week for 6 to 10 weeks [37, 41, 43, 45–48, 50]. Hypertensive participants were aged, men (*n* = 84) and women (*n* = 72) on a variety of BP medications. With all this in mind, hypertensives appear to experience large SBP reductions (weighted mean reduction of − 5.13 mmHg) following handgrip exercise training, further supporting the utility of handgrip exercise even among those taking pharmaceuticals. It should be noted that among all the data points collected for this review (including stratified data when available), the correlation between the pre-intervention group means in resting SBP and SBP reductions was not statistically significant, *r* = − 0.30, *p* = 0.07, *n* = 38.

Location of training also impacted training-induced resting SBP reductions such that research which required participants to complete exercise primarily in-laboratory under direct supervision of investigators [37, 38, 40, 41, 43, 44, 46, 48, 51, 53, 55–58, 61] (total participants, *n* = 230) reported greater SBP reductions (weighted mean, − 6.4 mmHg) than those which employed primarily at-home/unsupervised training regimens [38, 42, 45, 49, 50, 52] (total participants, *n* = 115) (weighted mean, − 2.6 mmHg). However, clinically relevant reductions of at least 2 mmHg [62] occurred regardless of exercise location, an impactful consideration when designing exercise programs with the fewest number of barriers to participation.

Finally, the impact of the exercise training stimulus (TTP) on SBP reduction was explored. In general, TTP mildly impacts the training response of SBP, as training prescriptions below the average of 473,540(seconds)(%MVC) resulted in a weighted mean reduction of − 6.2 mmHg (total participants, *n* = 299) while those above the average TTP resulted in a weighted mean reduction of − 3.6 mmHg (total participants, *n* = 167). It should be noted that there was no statistical correlation between TTP and SBP reduction, *r* = 0.22, *p* = 0.18.

#### Secondary outcomes and the impact of age and sex

Similar to SBP, weighted mean difference calculations for DBP reveal that handgrip training-induced DBP reductions are similar when merely comparing sexes (women, − 1.9 mmHg; men, − 1.2 mmHg) or ages (younger, − 2.5 mmHg; aged, − 1.8 mmHg) of diverse participants, with a potential advantage to the planned subcategory of aged women (− 2.4 mmHg). A summary of results can be found in Table 3.

Change in resting heart rate (HR (bpm)) as a result of handgrip exercise training was quantitatively reported in only 62% (*n* = 16) of the included literature, representing 278 total participants (women; at least 112). Of those, only nine had results that could be stratified for age and sex [38, 40, 42, 49, 51, 53, 58–60]. Results reveal that handgrip exercise training results in similarly small HR reductions when comparing sexes (women; − 0.9 bpm, men; − 0.6 bpm) or ages (younger, − 0.6 bpm; aged, − 0.3 bpm) of participants. A summary of results can be found in Table 4.

##### Arterial health and function

There were 13 studies (50%) that assessed the impact of handgrip training on arterial health and function by looking at wall-to-lumen ratio [58], brachial flow-mediated dilation (FMD) [42, 46, 51, 54, 55, 59], resting arterial diameter [39], pulse wave velocity [49, 50], and reactive hyperemic forearm blood flow [38, 51, 60]. This combined data represents 53 young men [54, 55, 59, 60], 38 young women [38, 42, 54, 55], 62 aged men [39, 42, 46, 50, 51], and 61 aged women [39, 46, 49, 50]. Handgrip training protocols were diverse with prescriptions of high intensity [39, 49, 51, 58–60], moderate intensity [42, 46, 50, 54, 55], rhythmic grips [39, 42, 49, 51, 58–60], and sustained grips [38, 46, 50, 54, 55]. Summary of results indicate that handgrip training generally improves arterial health and function, regardless of assessment variable. However, the results are not consistent with insignificant training effects on brachial FMD following a moderate intensity (40% MVC) intermittent training program among clinical patients requiring coronary angiography [42], insignificant training effects on measures of pulse wave velocity following low [50], moderate [50], and high intensity [49] handgrip training among aged participants, as well as insignificant training effects on brachial FMD following a moderate intensity handgrip training program among young otherwise healthy men and women [55].

##### Venous health and function

None of the collected literature assessed the impact of handgrip training on venous health and function.

##### Autonomic nervous function

There were nine studies (35%) that assessed the impact of handgrip training on autonomic nervous function by looking at MSNA [56], HRV [24, 38, 43, 47–49, 59], and/or cardiovascular reactivity [40, 45, 49]. It should be noted that some of the research represents data from mixed cohorts of both men and women which have not been stratified for sex analysis. Therefore, the data represents 92 young men and women [38, 40, 54, 56, 59] and 62 aged men and women [24, 43, 45, 47–49]. The vast majority of handgrip training protocols were moderate intensity with sustained grips with only two high intensity rhythmic grip protocols [24, 59]. All research evaluating young participants revealed insignificant effects of training on HRV [38, 59], MSNA [56], and cardiovascular reactivity [40]. Among aged participants there were mixed results with handgrip training causing either improvements to autonomic nervous function [24, 45, 47, 49] or no change to autonomic nervous function [43, 48]. Worth noting is that all six studies among aged participants included men and women taking various cardiovascular medications, which may account for the inconsistent results. Improvements to resting HRV were reported for the clinical population of coronary artery [47].

## Discussion

The purpose of this review was to systematically explore the impact of age and sex on the utility of handgrip exercise for resting SBP reduction. Using broad inclusion criteria and data provided by original researchers, this review is the first to segregate and evaluate the potential influence of participants’ age and sex, revealing that aged women may benefit the most from handgrip training protocols regardless of exercise prescription details. Moreover, it appears that clinically meaningful reductions in resting BP can be achieved regardless of the age or the sex of included participants.

### Deviations from the original review design

It was not possible to extract information on the timing of final assessment compared to final exercise bout due to limited reporting. Ideally, measures of resting BP should be at least 24 h to avoid overlapping results with post-exercise hypotension, yet less than 72 h to avoid effects of detraining.

The original research design proposed the deletion of all studies where sex of participants could not be segregated [16]. Due to the high volume of research conducted with mixed samples of men and women, combined with an inability to obtain segregated data from the original researchers, the decision was made to maintain the data of these studies in a third, “mixed men and women” category for data presentation. This data was included in the main calculations regarding the impact of age yet removed from the calculations regarding the impact of participant sex.

In the original research design, it was proposed that two assessment tools be used to assess study quality, the Quality Assessment Framework developed by the Cochrane Collaboration for randomized controlled trials and the Newcastle-Ottawa Scale for experimental exercise interventions without a designated control group. However, since this original research plan a new quality scale was developed specifically for exercise training: TESTEX. The TESTEX was designed to address the shortcomings of other quality assessment scales which include criteria which are not appropriate in exercise training studies [20]. Our TESTEX-based ratings (RCTs median, 8.0/15) are slightly less than those reported by Inder et.al. (median, 10/15) in their meta-analysis on the subject of isometric exercise for blood pressure reduction [11]. The original research design specified the removal of studies below a minimum threshold of quality. Upon further examination of the limited breadth of included literature it was decided that all identified studies would be included in this systematic review. For interest, there were three studies which scored less than 5/15, representing a total of 65 aged [37, 53] and young [36] men and women with a weighted mean SBP reduction of − 9.92 mmHg. Although in isolation, these three lower quality studies reflect a potential impact of study quality on SBP reduction, when the entire data set is examined there was an statistically insignificant correlation between study quality on resting SBP change scores, *r* = 0.02, *p* > 0.05.

Finally, the original review protocol outlined an assessment of bias across studies using the Grading of Recommendations Assessments, Development and Evaluation (GRADE) approach and heterogeneity calculations of *I*^2^ and its confidence interval. Unfortunately, only 2 studies reported the variance of the response score. As a result, we completed our planned back up approach which was to report change scores (Table 1) and complete a narrative synthesis of the presented data.

### Study quality assessment

Although a majority of studies had good study quality and study reporting (≥ 5 TESTEX scale; 23/26) consistent shortcomings among reporting were apparent. There were only two studies which reported blinding the research personal responsible for collecting physiological variables of interest [37, 39]. Furthermore, of the 12 RCT studies, only five (42%) reported the method of randomization [38, 41, 48, 52, 61] and only five (42%) attempted to monitor activity level of control group participants [38, 45, 48, 50, 55]. All of these factors are possible sources of bias that may have influenced study results. It is strongly recommended that future research consider such design characteristics when planning handgrip exercise interventions.

Furthermore, of the 11 studies reporting at least one participant drop-out, only two conducted an intention-to-treat analysis [40, 41]. Of note, there were an additional five studies that reported a dropout of zero removing the need to conduct such an analysis. Intention-to-treat analyses help to establish patterns of consistency between particular patient demographics, potentially identifying cohorts which are more likely to withdraw from exercise training studies [20].

### Primary variable of interest

The disparity in presentation of CVD between aged men and women is recognized worldwide, with the American Heart Association publishing separate guidelines for CVD prevention for men and women [63] and the European Society of Cardiology formally publishing sex-specific public policy on CVD [64]. With this growing recognition, it is imperative that research explore the influence of biological sex on the management of CVD risk, such as exercise interventions to reduce elevated BP. For this reason, our primary research aim was to compare the magnitude of resting SBP reduction between men and women following at least 4 weeks of handgrip exercise training. The results of this review found that on average women had a slightly greater reductions in resting SBP (− 5.6 mmHg) in comparison to men (− 4.4 mmHg). This impact is further pronounced with age, such that aged women (− 6.5 mmHg SBP reduction) appear to benefit more than aged men (− 4.7 mmHg SBP reduction).

Based on these findings, it appears that the magnitude of SBP reduction is dependent on biological sex, with greater reductions in women, with a potential interactive influence of age, leading to greater reductions with increasing age. Given the increased risk of CVD among aged women [4, 65], our findings provide support for the utilization of handgrip exercise to manage resting BP among post-menopausal women. Since handgrip can be done in any setting and is easy to complete, this type of training can overcome environmental and knowledge barriers to exercise that have been cited by older adults [66].

A clinically significant change in SBP, which corresponds to significant reductions in the incidence of cardiovascular disease among normotensive and hypertensive individuals, has been defined as a reduction by at least 2 mmHg [62]. Of the 26 studies included in this review, 16 studies had both statistically and clinically significant changes in SBP following the handgrip intervention [36–41, 43, 45–47, 49, 52–55, 57]. An additional six studies reported changes in SBP that were not statistically significant, but can be considered clinically significant [44, 51, 56, 59–61]. Clinically meaningful changes without statistical significance may reflect inadequate statistical power. Worth noting is that the sample size for the six statistically non-significant studies averaged 13 participants compared to 19.4 participants in studies demonstrating both statistical and clinical significance. Understanding the threshold for clinically significant results is critical when evaluating interventions for implementation into clinical practice. Considering that 85% of studies in this review reported clinically significant reductions in SBP, handgrip training appears to be an effective non-pharmaceutical strategy to manage BP for men and women, regardless of age.

Previous reviews on handgrip training have found various magnitudes of reduction in SBP. In a meta-analysis of randomized controlled trials by Kelley and Kelley [14], a large reduction of − 13.4 mmHg (95% bootstrap percentile CI: − 15.3 to − 11.0) was observed following handgrip training for at least 4 weeks. This reduction in SBP is over two times greater than the weighted mean differences calculated for men, women, young, and old participants collectively in this systematic review. The greater reduction in SBP may be due to the selective inclusion criteria resulting in the analysis of only three randomized controlled trials, whereas this systematic review had broad inclusion criteria with various study designs. In a more recent review by Inder et al. which included randomized controlled trials and cross-over studies, six studies with handgrip interventions were identified with a calculated SBP reduction of − 6.88 mmHg (95% CI − 8.31 to − 5.46) [11]. Our findings provide further support of handgrip exercise for resting BP reduction, with results that fall within this previously published range for both the cohort of women (young and old) (− 5.6 mmHg) and the young (men and women) (− 5.7 mmHg). Although our calculated results for men (young and old) (− 4.4 mmHg) and aged (men and women) (− 4.8 mmHg) are slightly below Inder et al.’s results, this slight variation in SBP reduction is likely due to the mixed-sex and mixed-age samples in the Inder et al. (2016) review. To our knowledge, our review is the first to segregate handgrip-induced reductions in BP based on both sex and age. As a result, this review provides some insight into determining age- and sex-specific advantages to handgrip training.

Although tangential to the primary objective of this systematic review, it was observed that in-lab/supervised training programs produced somewhat larger reductions in SBP (− 6.4 mmHg) compared to at-home/unsupervised training programs (− 2.6 mmHg). Interesting, this difference in training response may not be the result of training adherence, a commonly noted concern of unsupervised programs, as six of the eight at-home/unsupervised studies specifically reported adherence was ≥ 90%. Given the pragmatic importance of at-home training options for wide-spread promotion of healthy behaviors, further investigation is required in order to determine the reason for this location-specific difference in training response.

### Secondary variables of interest

DBP followed a similar trend to SBP with similar reductions for women (− 1.9 mmHg) and men (− 1.2 mmHg) along with comparable reductions among younger (− 2.5 mmHg) and aged (− 1.7 mmHg) study participants. Consistent with the SBP results, DBP reduction reveals that handgrip exercise training may be the most impactful for the sub-cohort of aged women that benefits the most from handgrip exercise training (− 2.4 mmHg reduction).

In the past, clinical attention was directed towards resting DBP values, as evidenced by the first JNC report which defined DBP as the basis for the detection and treatment of hypertension [67]. Given that there is little evidence to suggest significant risk of isolated diastolic hypertension [68], some clinicians have gone so far as to suggest that it not even be measured [69, 70]. The importance of DBP reduction for the maintenance of cardiovascular health remains an active conversation within the literature, with recent research showing clinical significance for DBP reduction for populations post-stroke [71]. Researchers employing handgrip exercise strategies are encouraged to continue to use DBP as a measured outcome variable so that we may continue to understand its clinical relevance.

The decision was made to include indicators of arterial and venous health and function in this systematic review as they have been previously proposed as potential mediating handgrip-induced reductions to systolic blood pressure. Surprisingly though, this review found that variables of vascular health and function were inconsistently measured within handgrip training studies. Of the research evaluating this variable, nearly all research which prescribed high intensity rhythmic handgrip reported significant improvements to vascular health including wall-to-lumen ratio [58], brachial and radial resting diameters [39], reactive hyperemic forearm blood flow [51, 60], brachial flow-mediated dilation [51, 59], vascular resistance [60], and peak shear rate [51]. These results are consistent with previous research showing that rhythmic handgrip exercise increased brachial arterial responses and peak reactive hyperemic blood flow among young men [72]. The basis for such improvements in vascular health may be linked to the repeated cyclical deviations to forearm blood flow characteristic of high-intensity intermittent handgrip exercise resulting in a shear stimulus large enough to mediate changes in vascular function and structure.

### Strengths and limitations

A noted strength of this review is the transparent approach, drawing on recommended and validated methods [17] with an accompanying review protocol [16]. In addition, the breadth of the study inclusion criteria has ensured that a comprehensive representation of handgrip training interventions has been presented. Finally, the use of two separate reviewers for the screening process, data abstraction, and quality appraisal increases the strength of conclusions.

A potential limitation of this review is the volume of included literature. Although handgrip exercise has been used for decades as a short-term stressor, the prescription of handgrip as a training modality to reduce BP is more recent. As researchers continue to use handgrip exercise as a training option for cardiovascular health, the breadth of literature for inclusion in future systematic reviews will increase.

Considering the various handgrip training prescriptions and devices used in the literature as well as the different participant samples, this review has a great deal of clinical heterogeneity. Variation in response to the intervention was infrequently reported. Consequently, we were unable to complete statistical analysis and quantitative integration of the data. A previous review calculated indices of heterogeneity based on a more restrictive set of studies (*n* = 10) and by assuming *p* values for studies that only reported above or below a critical *p* value [11].

### Recommendations for future research

Future handgrip training studies recruiting a mixed sample should consider reporting results separately for men and women as training responses may differ. In addition, since handgrip training is safe, effective, and time-efficient, it will be important to determine optimal handgrip training prescriptions prior to clinical implementation. Finally, further research exploring the potential mechanisms mediating the reduction in BP is warranted to better understand the physiological adaptations that occur with handgrip training. Such mechanisms include, but are not limited to, vascular indices of health and function as well as indices of autonomic nervous control. It may be worthwhile to determine if these adaptations are dependent on participants’ age and/or sex.

## Conclusion

In conclusion, this systematic review found that handgrip exercise training at least 4 weeks in duration was effective in reducing resting BP among men and women with various health statuses. Furthermore, it appears that on average the greatest reduction in resting BP occurs among aged women. Reductions in BP were comparable between younger (< 55 years) and aged (> 55 years) study participants. The findings of this review may be relevant for clinicians and individuals looking to manage BP through non-pharmaceutical interventions and may be helpful in informing future studies that seek to refine and implement handgrip training programs designed to best meet the needs of specific cohorts.

## Abbreviations

AMED: Allied and Contemporary Medicine
BP: Blood pressure
CINAHL: Cumulative Index to Nursing and Allied Health Literature
CV: Cardiovascular
CVD: Cardiovascular disease
DBP: Diastolic blood pressure
FMD: Flow-mediated dilation
GRADE: Grading of Recommendations Assessments, Development and Evaluation
HR: Heart rate
HRV: Heart rate variability
MAP: Mean arterial pressure
MSNA: Muscle sympathetic nerve activity
MVC: Maximal volitional contraction
PRISMA: Preferred Reporting Items for Systematic Review and Meta-Analysis
PROSPERO: Prospective Register of Systematic Reviews
RCT: Randomized controlled trials
SBP: Systolic blood pressure
TESTEX: Tool for the assEssment of Study qualiTy and reporting in EXercise
TTP: Total tension-time product

## References

[1] Organization WH (2017). Cardiovascular Diseases. Fact Sheet #317.

[2] Cornelissen VA, Fagard RH, Coeckelberghs E, Vanhees L (2011). Impact of resistance training on blood pressure and other cardiovascular risk factors: a meta-analysis of randomized, controlled trials. Hypertension.

[3] Organization WH (2013). Global Action Plan for the Preventation and Control of Noncommunicable Disease, 2013–2020.

[4] Wenger NK, Arnold A, Bairey Merz CN, Cooper-DeHoff RM, Ferdinand KC, Fleg JL, Gulati M, Isiadinso I, Itchhaporia D, Light-McGroary K (2018). Hypertension across a woman’s life cycle. J Am Coll Cardiol.

[5] Schenck-Gustafsson K (2009). Risk factors for cardiovascular disease in women. Maturitas.

[6] Yang XP, Reckelhoff JF (2011). Estrogen, hormonal replacement therapy and cardiovascular disease. Curr Opin Nephrol Hypertens.

[7] Lee SK, Khambhati J, Varghese T, Stahl EP, Kumar S, Sandesara PB, Wenger NK, Sperling LS (2017). Comprehensive primary prevention of cardiovascular disease in women. Clin Cardiol.

[8] Program CHE (2013). CHEP 2014 One Page Summary.

[9] Cornelissen VA, Smart NA (2013). Exercise training for blood pressure: a systematic review and meta-analysis. J Am Heart Assoc.

[10] Brook RD, Appel LJ, Rubenfire M, Ogedegbe G, Bisognano JD, Elliott WJ, Fuchs FD, Hughes JW, Lackland DT, Staffileno BA (2013). Beyond medications and diet: alternative approaches to lowering blood pressure: a scientific statement from the American Heart Association. Hypertension.

[11] Inder JD, Carlson DJ, Dieberg G, McFarlane JR, Hess NC, Smart NA (2016). Isometric exercise training for blood pressure management: a systematic review and meta-analysis to optimize benefit. Hypertens Res.

[12] McGowan CL, Proctor DN, Swaine I, Brook RD, Jackson EA, Levy PD (2017). Isometric handgrip as an adjunct for blood pressure control: a primer for clinicians. Curr Hypertens Rep.

[13] Owen A, Wiles J, Swaine I (2010). Effect of isometric exercise on resting blood pressure: a meta analysis. J Hum Hypertens.

[14] Kelley GA, Kelley KS (2010). Isometric handgrip exercise and resting blood pressure: a meta-analysis of randomized controlled trials. J Hypertens.

[15] Carlson DJ, Dieberg G, Hess NC, Millar PJ, Smart NA (2014). Isometric exercise training for blood pressure management: a systematic review and meta-analysis. Mayo Clin Proc.

[16] Bentley DC, Nguyen CH, Thomas SG (2015). Resting blood pressure reductions following isometric handgrip exercise training and the impact of age and sex: protocol for a systematic review. Syst Rev.

[17] Moher D, Liberati A, Tetzlaff J, Altman DG, Group P (2009). Preferred reporting items for systematic reviews and meta-analyses: the PRISMA statement. J Clin Epidemiol.

[18] Gold EB (2011). The timing of the age at which natural menopause occurs. Obstet Gynecol Clin N Am.

[19] Aging NIo (2013). AgePage - Menopause.

[20] Smart NA, Waldron M, Ismail H, Giallauria F, Vigorito C, Cornelissen V, Dieberg G (2015). Validation of a new tool for the assessment of study quality and reporting in exercise training studies: TESTEX. Int J Evid Based Healthc.

[21] Lin S, Chen Y, Li Y, Li J, Lu X (2014). Physical ischaemia induced by isometric exercise facilitated collateral development in the remote ischaemic myocardium of humans. Clin Sci (Lond).

[22] Wimer GS, Baldi JC (2012). Limb-specific training affects exercise hyperemia but not sympathetic vasoconstriction. Eur J Appl Physiol.

[23] Green DJ, Cable NT, Fox C, Rankin JM, Taylor RR (1994). Modification of forearm resistance vessels by exercise training in young men. J Appl Physiol (1985).

[24] Piepoli M, Clark AL, Volterrani M, Adamopoulos S, Sleight P, Coats AJ (1996). Contribution of muscle afferents to the hemodynamic, autonomic, and ventilatory responses to exercise in patients with chronic heart failure: effects of physical training. Circulation.

[25] Ash GI, Taylor BA, Thompson PD, MacDonald HV, Lamberti L, Chen MH, Farinatti P, Kraemer WJ, Panza GA, Zaleski AL (2017). The antihypertensive effects of aerobic versus isometric handgrip resistance exercise. J Hypertens.

[26] McGowan CL, Levy AS, Millar PJ, Guzman JC, Morillo CA, McCartney N, Macdonald MJ (2006). Acute vascular responses to isometric handgrip exercise and effects of training in persons medicated for hypertension. Am J Physiol Heart Circ Physiol.

[27] Bartol C, Kenno K, McGowan C (2012). Post-exercise hypotension: effects of acute and chronic isometric handgrip in well-controlled hyppertensives. Crit Rev Phys Rehab Med.

[28] Zhang J (2003). Effect of isometric handgrip exercise training on resting hemodynamics: a pilot study. J Chiropr Med.

[29] Millar PJ, Bray SR, McGowan CL, MacDonald MJ, McCartney N (2007). Effects of isometric handgrip training among people medicated for hypertension: a multilevel analysis. Blood Press Monit.

[30] Smart NA, Carlson DJ, Swaine I, McGowan C (2017). Commentary on aerobic versus isometric handgrip exercise in hypertension: a randomized controlled trial. J Hypertens.

[31] Shammas RA, Mullen K, Chuang PP, Bank AJ (1998). Effects of short-term forearm exercise training on resistance vessel endothelial function in normal subjects and patients with heart failure. J Card Fail.

[32] Katz SD, Yuen J, Bijou R, LeJemtel TH (1997). Training improves endothelium-dependent vasodilation in resistance vessels of patients with heart failure. J Appl Physiol (1985).

[33] Mostoufi-Moab S, Widmaier EJ, Cornett JA, Gray K, Sinoway LI (1998). Forearm training reduces the exercise pressor reflex during ischemic rhythmic handgrip. J Appl Physiol (1985).

[34] Sinoway L, Shenberger J, Leaman G, Zelis R, Gray K, Baily R, Leuenberger U (1996). Forearm training attenuates sympathetic responses to prolonged rhythmic forearm exercise. J Appl Physiol (1985).

[35] Somers VK, Leo KC, Shields R, Clary M, Mark AL (1992). Forearm endurance training attenuates sympathetic nerve response to isometric handgrip in normal humans. J Appl Physiol (1985).

[36] Garg R, Malhotra V, Kumar A, Dhar U, Tripathi Y (2014). Effect of isometric handgrip exercise training on resting blood pressure in normal healthy adults. J Clin Diagn Res.

[37] Peters PG, Alessio HM, Hagerman AE, Ashton T, Nagy S, Wiley RL (2006). Short-term isometric exercise reduces systolic blood pressure in hypertensive adults: possible role of reactive oxygen species. Int J Cardiol.

[38] Badrov MB, Bartol CL, DiBartolomeo MA, Millar PJ, McNevin NH, McGowan CL (2013). Effects of isometric handgrip training dose on resting blood pressure and resistance vessel endothelial function in normotensive women. Eur J Appl Physiol.

[39] Kumar S, Seward J, Wilcox A, Torella F (2010). Influence of muscle training on resting blood flow and forearm vessel diameter in patients with chronic renal failure. Br J Surg.

[40] Somani Y, Baross A, Levy P, Zinszer K, Milne K, Swaine I, McGowan C (2017). Reductions in ambulatory blood pressure in young normotensive men and women after isometric resistance training and its relationship with cardiovascular reactivity. Blood Press Monit.

[41] Carlson DJ, Inder J, Palanisamy SK, McFarlane JR, Dieberg G, Smart NA (2016). The efficacy of isometric resistance training utilizing handgrip exercise for blood pressure management: a randomized trial. Medicine (Baltimore).

[42] Dawson EA, Alkarmi A, Thijssen DH, Rathore S, Marsman DE, Cable NT, Wright DJ, Green DJ (2012). Low-flow mediated constriction is endothelium-dependent: effects of exercise training after radial artery catheterization. Circ Cardiovasc Interv.

[43] Taylor AC, McCartney N, Kamath MV, Wiley RL (2003). Isometric training lowers resting blood pressure and modulates autonomic control. Med Sci Sports Exerc.

[44] Baross AW, Hodgson DA, Padfield SL, Swaine IL (2017). Reductions in resting blood pressure in young adults when isometric exercise is performed whilst walking. J Sports Med (Hindawi Publ Corp).

[45] Badrov MB, Horton S, Millar PJ, McGowan CL (2013). Cardiovascular stress reactivity tasks successfully predict the hypotensive response of isometric handgrip training in hypertensives. Psychophysiology.

[46] McGowan CL, Visocchi A, Faulkner M, Verduyn R, Rakobowchuk M, Levy AS, McCartney N, MacDonald MJ (2007). Isometric handgrip training improves local flow-mediated dilation in medicated hypertensives. Eur J Appl Physiol.

[47] Millar PJ, Levy AS, McGowan CL, McCartney N, MacDonald MJ (2013). Isometric handgrip training lowers blood pressure and increases heart rate complexity in medicated hypertensive patients. Scand J Med Sci Sports.

[48] Stiller-Moldovan C, Kenno K, McGowan CL (2012). Effects of isometric handgrip training on blood pressure (resting and 24 h ambulatory) and heart rate variability in medicated hypertensive patients. Blood Press Monit.

[49] Bentley DC, Nguyen CHP, Thomas SG (2018). High-intensity handgrip training lowers blood pressure and increases heart rate complexity among postmenopausal women: a pilot study. Blood Press Monit.

[50] Pagonas N, Vlatsas S, Bauer F, Seibert FS, Zidek W, Babel N, Schlattmann P, Westhoff TH (2017). Aerobic versus isometric handgrip exercise in hypertension: a randomized controlled trial. J Hypertens.

[51] Dobrosielski DA, Greenway FL, Welsh DA, Jazwinski SM, Welsch MA, Louisiana Healthy Aging S (2009). Modification of vascular function after handgrip exercise training in 73- to 90-yr-old men. Med Sci Sports Exerc.

[52] Goessler KF, Buys R, VanderTrappen D, Vanhumbeeck L, Cornelissen VA (2018). A randomized controlled trial comparing home-based isometric handgrip exercise versus endurance training for blood pressure management. J Am Soc Hypertens.

[53] Millar PJ, Bray SR, MacDonald MJ, McCartney N (2008). The hypotensive effects of isometric handgrip training using an inexpensive spring handgrip training device. J Cardiopulm Rehabil Prev.

[54] Badrov MB, Freeman SR, Zokvic MA, Millar PJ, McGowan CL (2016). Isometric exercise training lowers resting blood pressure and improves local brachial artery flow-mediated dilation equally in men and women. Eur J Appl Physiol.

[55] McGowan CL, Levy AS, McCartney N, MacDonald MJ (2007). Isometric handgrip training does not improve flow-mediated dilation in subjects with normal blood pressure. Clin Sci (Lond).

[56] Ray CA, Carrasco DI (2000). Isometric handgrip training reduces arterial pressure at rest without changes in sympathetic nerve activity. Am J Physiol Heart Circ Physiol.

[57] Wiley RL, Dunn CL, Cox RH, Hueppchen NA, Scott MS (1992). Isometric exercise training lowers resting blood pressure. Med Sci Sports Exerc.

[58] Thijssen DH, Dawson EA, van den Munckhof IC, Tinken TM, den Drijver E, Hopkins N, Cable NT, Green DJ (2011). Exercise-mediated changes in conduit artery wall thickness in humans: role of shear stress. Am J Physiol Heart Circ Physiol.

[59] Allen JD, Geaghan JP, Greenway F, Welsch MA (2003). Time course of improved flow-mediated dilation after short-term exercise training. Med Sci Sports Exerc.

[60] Alomari MA, Mekary RA, Welsch MA (2010). Rapid vascular modifications to localized rhythmic handgrip training and detraining: vascular conditioning and deconditioning. Eur J Appl Physiol.

[61] Hess NC, Carlson DJ, Inder JD, Jesulola E, McFarlane JR, Smart NA (2016). Clinically meaningful blood pressure reductions with low intensity isometric handgrip exercise**.** A randomized trial. Physiol Res.

[62] Turnbull F, Blood Pressure Lowering Treatment Trialists C (2003). Effects of different blood-pressure-lowering regimens on major cardiovascular events: results of prospectively-designed overviews of randomised trials. Lancet.

[63] Mosca L, Benjamin EJ, Berra K, Bezanson JL, Dolor RJ, Lloyd-Jones DM, Newby LK, Pina IL, Roger VL, Shaw LJ (2011). Effectiveness-based guidelines for the prevention of cardiovascular disease in women--2011 update: a guideline from the American Heart Association. Circulation.

[64] Stramba-Badiale M, Fox KM, Priori SG, Collins P, Daly C, Graham I, Jonsson B, Schenck-Gustafsson K, Tendera M (2006). Cardiovascular diseases in women: a statement from the policy conference of the European Society of Cardiology. Eur Heart J.

[65] Lerner DJ, Kannel WB (1986). Patterns of coronary heart disease morbidity and mortality in the sexes: a 26-year follow-up of the Framingham population. Am Heart J.

[66] Schutzer KA, Graves BS (2004). Barriers and motivations to exercise in older adults. Prev Med.

[67] The fifth report of the Joint National Committee on Detection, Evaluation, and treatment of high blood pressure (JNC V). Arch Intern Med. 1993;153(2):154–83. 8422206

[68] Tin LL, Beevers DG, Lip GY (2002). Systolic vs diastolic blood pressure and the burden of hypertension. J Hum Hypertens.

[69] Swales JD (2000). Systolic versus diastolic pressure: paradigm shift or cycle?. J Hum Hypertens.

[70] Sever P (1999). Abandoning diastole. BMJ.

[71] Katsanos AH, Filippatou A, Manios E, Deftereos S, Parissis J, Frogoudaki A, Vrettou AR, Ikonomidis I, Pikilidou M, Kargiotis O (2017). Blood pressure reduction and secondary stroke prevention: a systematic review and metaregression analysis of randomized clinical trials. Hypertension.

[72] Tinken TM, Thijssen DH, Hopkins N, Dawson EA, Cable NT, Green DJ (2010). Shear stress mediates endothelial adaptations to exercise training in humans. Hypertension.

